# Mind the treatment gap: the prevalence of common mental disorder symptoms, risky substance use and service utilization among young Swiss adults

**DOI:** 10.1186/s12889-020-09577-6

**Published:** 2020-09-29

**Authors:** Laura Werlen, Milo A. Puhan, Markus A. Landolt, Meichun Mohler-Kuo

**Affiliations:** 1 La Source, School of Nursing Sciences, HES-SO University of Applied Sciences and Arts of Western Switzerland, Lausanne, Switzerland; 2 Department of Child and Adolescent, Psychiatry and Psychotherapy, University Hospital of Psychiatry Zurich, University of Zurich, Zurich, Switzerland; 3 Epidemiology, Biostatistics and Prevention Institute, University of Zurich, Zurich, Switzerland; 4 Department of Psychosomatics and Psychiatry, University Children’s Hospital Zurich, Zurich, Switzerland; 5 Department of Psychology, Division of Child and Adolescent Health Psychology, University of Zurich, Zurich, Switzerland

**Keywords:** Adolescents, Young adult, Mental disorders, Anxiety disorders, Depression, Attention deficit hyperactivity disorder, Alcohol consumption, Drug utilization, Mental health services, Service utilization

## Abstract

**Background:**

Common mental disorders (CMDs) are highly prevalent and contribute significantly to the global burden of disease, yet there is evidence of a large treatment gap. We aimed to quantify this gap among young adults with symptoms of CMDs and examine the relationship between substance use and perceived need for care and mental health service utilization.

**Methods:**

In a nationally representative, cross-sectional survey of young Swiss adults’ mental health and wellbeing, we assessed symptoms of anxiety, depression, and attention deficit hyperactivity disorder (ADHD) with widely used screening instruments and asked about participant suicidal ideation, suicide attempts, mental health-related quality of life, alcohol and drug use, perceived need for mental health care, and mental health service utilization. We used these variables to calculate the treatment gap and weighted all analyses according to the stratified sampling plan.

**Results:**

Around a quarter of young adults screened positive for at least one CMD. Participants who screened positive for anxiety and/or depression reported significantly more suicidal ideation and lifetime suicide attempts and reported worse mental health-related quality of life than participants who did not screen positive for a disorder. Women’s prevalence of anxiety and depression symptoms was significantly higher than men’s, while men were more likely to report most types of risky drug use. Among those with a CMD, only around half perceived lifetime need for care, and less than 20% reported currently utilizing mental health services. Young adults with a CMD reporting risky weekly use of alcohol were less likely to be currently using services.

**Conclusion:**

The high prevalence of CMD symptoms could reflect a rising prevalence of these disorders mirroring increasing trends observed in other countries. To address the large treatment gap, interventions promoting mental health literacy and more research on additional barriers to inform further interventions are needed.

## Background

Throughout the world, common mental disorders (CMDs), which include anxiety-related and depressive disorders [1], are highly prevalent. An estimated 4.4% of the world’s population suffers from depression alone in a given year [1]. In addition, CMDs constitute one of the leading causes of disability-adjusted life years (DALYs) [2], and CMDs’ global burden continues to grow over time [3]: by 2030, depression is predicted to become the leading cause of disability in high-income countries [4]. Though the onset of mental disorders varies, most typically have their onset in late adolescence [5], and the burden of these illnesses is especially high for young people [3]. In Switzerland, the burden and costs are also significant; the accompanying total economic costs have been estimated at 3.5% of GDP [6].

Despite the significant impact of CMDs, there is evidence that many people suffering from such disorders are never diagnosed or do not access timely and/or appropriate treatment; across the world, this treatment gap has been estimated at over 50%, meaning that more than half of the people who could benefit from services do not reach them [7, 8]. Even among severe cases of mental disorders, only around half ever receive mental health treatment [9].

Expanding beyond estimates of prevalence and service utilization by quantifying and assessing the treatment gap is important in order to better understand who is not reaching services and why. In addition, the consequences of not receiving professional care need to be measured, including potential self-medication through the risky use of alcohol and drugs [10]. Previous research on factors impacting service utilization shows that barriers are wide-ranging, from individual behaviors to social norms to environmental factors [11, 12]. Young adults face specific obstacles related to their stage of life (e.g., limited financial resources, unfamiliarity with the health care system, fear of judgment from family, peers, or employers) [10, 13]. Certain sub-groups of the population, such as people of low socio-encomic status or ethnic minorities, must contend with even more barriers [12].

Despite the dimensions and importance of the burden of CMDs on young adults, there are hardly any epidemiological data on the prevalence of mental disorders, service utilization, and therefore the treatment gap in Switzerland as yet, and this is especially the case for young people [14]. In the context of international trends indicating the growing burden and increased prevalence of CMDs [15, 16], we aimed to estimate the prevalence of the CMDs depression and anxiety as well as ADHD (another one of the most prevalent disorders in adolescence and young adulthood [17–19]), risky substance use and the prevalence of perceived need for treatment, lifetime and current service utilization, and the treatment gap between those in need of professional health services and those who receive them among those diagnosed using a representative sample of young adults in Switzerland. To explore possible causes of the treatment gap, we also analyzed whether participants with CMDs who reported risky substance use were less likely to use mental health services.

## Methods

### Study design

#### Participants

We used data from the Swiss Youth Epidemiological Study on Mental Health (S-YESMH), a nationally representative, cross-sectional study of Swiss young adults’ mental health and wellbeing that was funded by the Swiss National Science Foundation and approved by the Ethics Committee of the Canton of Vaud (2017–00522). In order to obtain answers from participants of both Swiss and Non-Swiss nationality from all states (cantons), we developed a sampling plan stratified by canton, sex, and Swiss nationality that oversampled Non-Swiss participants, for which we adjusted in our statistical analyses. Based on this plan, the Swiss Federal Statistical Office provided us a random sample of 9805 young adults legally residing in Switzerland born between 1996 and 2000 (17–21 years old on December 31, 2017) who were randomly selected from the population register within each stratum specified by the sampling plan. Participants who were not able to complete the survey in German, French, or Italian were excluded from the survey. From the 9286 valid addresses in the sample at the time of data collection, 3840 participants (41.4%) completed the online survey.

#### Procedure

The data were collected via a cross-sectional online survey from February to August 2018 by the market research organization LINK Institute (**www.link.ch**). We sent an invitation letter by postal mail to every person sampled. This letter described the study, stated that the survey was voluntary and that answers would never be linked to participant contact information, and provided free hotline numbers for participants to contact the study team, LINK Institute, or mental health organizations if they had additional questions about the study or mental health in general. Participants could access the survey by entering the web address and password provided or by scanning an individualized QR code on the invitation letter. The survey duration was around 25 min and included questions on socio-demographic characteristics, symptoms of several common psychological disorders and suicide, somatic symptoms, service use, quality of life, sources of stress, social support, and alcohol and drug use. After three weeks, a first reminder letter was sent to participants who had not responded to the survey. Two weeks after this, LINK Institute called those who had not participated to encourage them to participate. Those who provided their e-mail address during the telephone call, but had not filled out the survey, were reminded to participate by e-mail. We sent a second reminder letter that included an optional paper version of the questionnaire one month after the telephone reminders began. Finally, we sent a last reminder letter four months after sending the initial invitation letter.

### Measures

#### Symptoms of common mental disorders (CMDs)

The common mental disorders in focus in this study were depression, anxiety, and attention deficit hyperactivity disorder (ADHD). Two Patient Health Questionnaire (PHQ) screeners were used to assess symptoms of CMDs: the Generalized Anxiety Disorder 7 (GAD-7) for anxiety [20] and the PHQ-9 for depression [21]. Both are widely used in clinical settings and have been validated in populations across the world [22–24] (note: though the German, French, and Italian versions have been validated, they have not all been validated in Switzerland specifically). For the PHQ-9, sensitivity is 80% and specificity is 92%, while the sensitivity of the GAD-7 is 89% and its specificity is 82% [23]. In this study, Cronbach’s alpha was 0.85 for the PHQ-9 and 0.87 for the GAD-7. These screeners ask about symptoms of anxiety and depression, respectively, over the past two weeks using a four-point rating scale for which 0 indicates ‘not at all’ and 3 indicates ‘always’. Based on the literature, we dichotomized total scores for these screeners into moderate anxiety and above (scores greater than or equal to 10) and moderate depression and above (scores greater than or equal to 10) [23]. ADHD symptoms were assessed using the Adult ADHD Self-Report Scale Screener (ASRS-v1.1), a validated six-item instrument about symptoms of ADHD during the past 6 months [25–27]. This instrument has adequate sensitivity (68.7%) and is highly specific (99.5%) [25]. Cronbach’s alpha was 0.6 in this study. We dichotomized total scores into ‘no ADHD’ (scores below 14) and ‘ADHD’ (scores 14–24) [28]. We also created a variable ‘anxiety, depression, or ADHD’ that included any participant who screened positive for at least one of these disorders.

Because the instruments used to measure CMDs assessed self-reported symptoms, we do not have sufficient information to establish a formal clinical diagnosis. In this study, we thus refer to people who report symptoms of the CMDs under study and probably suffer from them.

#### Risky substance use

We assessed risky alcohol and substance use with questions about the usual quantity and frequency of substance consumption using the questionnaire from the Cohort Study on Substance Use Risk Factors (C-SURF), a study of young men in Switzerland [29, 30]. Risky alcohol use was defined as the frequency of risky single-occasion drinking over the last 12 months. For men, risky single-occasion drinking meant consuming at least six standard drinks on a single occasion, and for women, four standard drinks (the definition of a standard drink was clarified using pictures with examples). We calculated two dichotomous variables for risky alcohol use: risky alcohol use at least monthly and risky alcohol use at least weekly based on the frequency of risky single-occasion drinking in each time period. Risky drug use was calculated for different classes of illicit substances. Risky cannabis use was defined as using cannabis at least 2–3 times per week; risky use of non-prescribed prescription drugs was defined as using prescription drugs not prescribed by a doctor at least two to three times per year; and risky illicit drug use was defined as using any other illicit drug at least four times throughout the participant’s lifetime.

#### Other mental health outcomes

Current suicidal ideation was measured using the question from the PHQ-9 “How often have you been bothered by thoughts that you would be better off dead or of hurting yourself in some way over the last two weeks?” Answers were dichotomized into no suicidal ideation (answer choice “not at all”) and suicidal ideation (combined answer choices “several days,” “more than half the days,” and “nearly every day”). We assessed lifetime suicide attempts using the question from the Swiss Health Survey 2017 “Have you ever attempted to take your life?” [31].

Mental health-related quality of life was measured using the Short-Form 12 Health Survey, Version 2 (SF-12) [32]. We calculated the Mental Component Summary score following the standard procedure using norm-based methods described in the SF-12 user manual. A score of 50 represents the average for the U.S. population in 1998, with higher scores indicating better quality of life.

#### Perceived need for and utilization of mental health care services

Questions on perceived need for health and mental health care service utilization were assessed using questions modified from the World Health Organization World Mental Health Composite International Diagnostic Interview (WHO WMH-CIDI) [33]. Lifetime perceived need for health care services was assessed by the yes/no question “Has there ever been a time in your life that you believe you required help for problems with your emotions, nerves, mental health, or your use of alcohol or drugs?” Lifetime mental health care service utilization was assessed by asking whether the participant had ever spoken with a health professional (i.e., psychiatrist, general practitioner, nurse, psychologist, care personnel, or counselor) in person or on the phone for problems with emotions, nerves, or use of alcohol or drugs. For current mental health care service utilization, we asked the same question for the period including the last four weeks.

#### Treatment gap

To measure the treatment gap, we first identified those who screened positive for CMDs and then measured their use of mental health care services by asking those who reported a perceived lifetime need for mental health care services a yes/no question about whether this need had been met. In addition, we looked at the percentage of participants who met the criteria for a disorder, yet had not used health care services either in the last four weeks or at any point during their lifetime.

#### Service availability

Service availability was represented by the regional psychiatrist density, which we defined as the number of psychiatrists per 100,000 inhabitants in the seven large Nomenclature of Territorial Units for Statistics 2 (NUTS-2) regions defined by the Swiss Federal Statistical Office (i.e., Lake Geneva region, Espace Mittelland, Northwestern Switzerland, Zurich, Eastern Switzerland, Central Switzerland, and Ticino) [34, 35].

#### Income and other socio-demographic variables

We asked participants about their approximate monthly household income level. Less than 6000 CHF was classified as low, around 6000 CHF was classified as middle, and more than 6000 CHF was classified as high for Swiss households. All other answers were classified as unknown. The Swiss Federal Statistical Office provided all other socio-demographic variables, including age, sex, nationality, and language region.

### Statistical analyses

All statistical analyses were conducted using R version 3.4.2 [36] and SPSS version 25 [37]. We adjusted our estimates to account for the stratified sampling design described above and non-response using the R package “survey” [38]. We used the package to specify our survey design. The sampling probabilities were computed for simple random sampling within strata as specified by the sampling plan. The true population sizes for each stratum were provided by the Swiss Federal Statistical Office. Because the percentage of missing values was limited to less than 2% for all variables, we did not impute any values.

To describe the study participants, we calculated weighted mean values and proportions for age, sex, Swiss nationality, and language region as well as the prevalence of CMDs, risky substance use, perceived need for help, and professional mental health service utilization (lifetime and current) by sex and Swiss nationality. We compared the mental health outcomes current suicidal ideation, lifetime suicide attempts, and mental health-related quality of life between participants who did and did not screen positive for one or more CMDs.

To calculate the treatment gap, we used these estimates to determine the percentage of participants who screened positive for CMDs who either did not perceive a need for care or had not used professional mental health services.

We examined the association of risky alcohol and drug use with perceived need for care and service utilization by performing logistic regression analyses for each drug individually, first alone and then adjusting for relevant socio-demographic and health care service characteristics.

## Results

### Participant socio-demographic characteristics

We used a weighting procedure to adjust our results to reflect the socio-demographic characteristics of the Swiss population (Table 1). After weighting, the average age of the participants was 19.6 years, 49.5% were female, 81.3% had Swiss nationality, and 68.3% were from the German-speaking region, 27.1% from the French-speaking region, and 4.7% from the Italian-speaking region. These weighted values fairly closely represented the socio-demographic characteristics of the population [35].

**Table 1:** Demographic characteristics of participants (*n* = 3840)

	Unweighted	Weighted
Mean age in years	19.6 (95% CI: 19.5–19.6)	19.6 (95% CI: 19.5–19.6)
Sex (% female)	57.7% (95% CI: 56.1–59.3)	49.5% (95% CI: 49.0–50.0)
Nationality (% Swiss)	77.1% (95% CI: 75.8–78.5)	81.3% (95% CI: 81.2–81.4)
Region (%)		
German-speaking	65.4% (95% CI: 63.9–66.9)	68.3% (95% CI: 67.7–68.9)
French-speaking	28.6% (95% CI: 27.2–30.1)	27.1% (95% CI: 26.5–27.6)
Italian-speaking	6.0% (95% CI: 5.3–6.8)	4.7% (95% CI: 4.4–4.9)

### Prevalence of CMD symptoms and risky substance use

Our study found that a quarter of youth reported symptoms indicating the presence of at least one of the CMDs (Table 2). Among these disorders, the most prevalent was depression at 17.7% followed by anxiety (13.2%) and ADHD (8.7%). The prevalence estimates for anxiety, depression, and screening positive for any of the three disorders were significantly higher in women than for men; for ADHD, the estimates were also higher, though not significantly so. The same pattern was found among Non-Swiss as opposed to Swiss participants (29.5% vs. 23.6% for any of the three disorders, 11.2% vs. 8.1% for ADHD, 16.5% vs. 12.4% for anxiety, and 23.1% vs. 16.4% for depression).

**Table 2:** Weighted prevalence of common mental disorders and risky substance use by sex and nationality (*n* = 3840)

	Total	Male	Female	Swiss	Non-Swiss
	% (95% CI)	% (95% CI)	% (95% CI)	% (95% CI)	% (95% CI)
ADHD, anxiety, or depression^a,b^	24.7% (23.3–26.1)	18.8% (16.9–20.8)	30.7% (28.8–32.7)	23.6% (22.1–25.2)	29.5% (26.2–32.7)
ADHD	8.7% (7.7–9.6)	7.4% (6.1–8.8)	9.9% (8.6–11.2)	8.1% (7.1–9.0)	11.2% (9.0–13.5)
Anxiety^a^	13.2% (12.1–14.2)	9.1% (7.7–10.6)	17.3% (15.7–18.9)	12.4% (11.2–13.6)	16.5% (13.9–19.1)
Depression^a,b^	17.7% (16.5–18.9)	11.5% (9.9–13.1)	23.9% (22.1–25.8)	16.4% (15.1–17.7)	23.1% (20.2–26.1)
Risky alcohol use^b^ (at least monthly)	36.2% (34.7–37.8)	37.6% (35.2–40.0)	34.8% (32.8–36.8)	39.6% (37.8–41.4)	21.7% (18.8–24.6)
Risky alcohol use^a,b^ (at least weekly)	11.2% (10.1–12.2)	13.0% (11.4–14.7)	9.3% (8.1–10.5)	12.3% (11.1–13.5)	6.5% (4.7–8.3)
Risky cannabis use^a^	6.2% (5.4–7.0)	8.5% (7.1–9.9)	3.9% (3.0–4.7)	6.5% (5.5–7.4)	5.1% (3.5–6.7)
Risky illicit drug use^a^	3.8% (3.2–4.4)	4.8% (3.8–5.9)	2.8% (2.0–3.5)	4.0% (3.3–4.8)	2.8% (1.6–4.0)
Risky non-prescribed prescription drug use^a^	9.7% (8.8–10.7)	7.7% (6.4–9.1)	11.8% (10.4–13.2)	9.8% (8.7–10.9)	9.5% (7.5–11.6)

^a^Significantly different between male and female; ^b^ Significantly different between Swiss and non-Swiss

Over one-third of participants reported risky alcohol use at least monthly; risky alcohol use at least weekly was 11.2%. Risky cannabis use was 6.2%, risky illicit drug use was 3.8%, and risky non-prescribed prescription drug use was 9.7%. While women reported lower weekly risky alcohol use and risky use of cannabis and illicit drugs, men and women did not differ in monthly risky use of alcohol, and women reported more risky use of non-prescribed prescription drugs. Compared with Non-Swiss participants, Swiss participants were significantly more likely to report both monthly and weekly risky alcohol use; however, risky substance use did not otherwise significantly differ between Swiss and Non-Swiss.

### Relationship between positive CMD screening and other mental health outcomes

Screening positive for one or more CMD was generally associated with more current suicidal ideation, lifetime suicide attempts, and worse mental health-related quality of life (Table 3). The only CMD that was not associated with worse mental health-related quality of life than that of those who did not screen positive for a CMD was ADHD alone. Screening positive for depression alone was associated with worse mental health outcomes than anxiety alone. The more comorbid positive CMD screenings, the more current suicidal ideation and lifetime suicide attempts and the worse mental health-related quality of life.

### Perceived need for care, service utilization, and treatment gap

The proportion of participants who perceived a need for care and used mental health care services differed by CMD and number of comorbidities (Table 4). Participants who screened positive for ADHD alone had the lowest perceived need and service utilization, while participants with all three diagnoses had the highest (comparing the two groups, one-third vs. two-thirds perceived a need for care, a quarter vs. just over half had ever used services, and less than 10% vs. almost one-third were currently using services). Among participants who screened positive for at least one CMD, almost half did not perceive a need for treatment, almost two-thirds had not ever utilized professional health care services during their lifetime, and more than four in five were not currently using professional health care services.

**Table 3:** Relationship between screening positive for common mental disorders and other health-related outcomes (*n* = 3840, weighted analysis)

	Prevalence of current suicidal ideation	Prevalence of lifetime suicide attempt	Mental health-related quality of life
	% (95% CI)	% (95% CI)	Mean (95% CI)
No diagnosis	5.1% (4.3–6.0)	2.1% (1.5–2.6)	56.6 (56.3–57.0)
ADHD alone	6.4% (1.9–10.8)	3.9% (0.0–7.4)	51.1 (48.9–53.4)
Anxiety alone	17.1% (10.1–24.2)	5.3% (1.4–9.3)	44.6 (42.7–46.6)
Depression alone	41.9% (35.6–48.1)	10.7% (6.7–14.7)	39.9 (38.3–41.5)
Anxiety and ADHD	23.4% (3.4–43.5)	–^b^	43.4 (38.4–48.4)
Depression and ADHD	39.1% (25.9–52.3)	5.3% (0.0-11.4)^a^	40.6 (37.0–44.3)
Anxiety and depression	51.0% (44.6–57.5)	19.6% (14.5–24.8)	34.9 (33.5–36.4)
ADHD, anxiety, and depression	63.2% (54.8–71.7)	28.0% (20.0–36.1)	30.7 (28.8–32.6)
Anxiety, depression, or ADHD	38.6% (35.4–41.8)	13.0% (10.8–15.2)	39.7 (38.8–40.6)

^a^Confidence interval rounded to stay within possible values ^*b*^*no estimate*

As we found for the prevalence rates of CMD symptoms, there were sex and nationality differences in perceived need for care and service utilization. Among those screening positive for CMDs, a higher percentage of women tended to perceive a need for care; however, it should be noted that the confidence intervals for these point estimates are relatively wide and overlapping between women and men. In general, the same pattern was observed for service utilization. Non-Swiss participants reported similar levels of perceived need for care as Swiss participants with the exception of those who screened positive for anxiety alone, anxiety and ADHD, and depression and ADHD, who all reported less perceived need for care. For anxiety alone and anxiety and ADHD, we observed the same pattern for lifetime service utilization; however, when assessing current service utilization, we observed the opposite pattern for anxiety alone (i.e., a higher percentage of non-Swiss participants reported currently using services).

### Association of alcohol and drug use with perceived need for care and mental health care service utilization

As opposed to participants who screened positive for a CMD and reported risky alcohol use, those who reported risky use of cannabis, illicit drugs, or non-prescribed prescription drugs were more likely to perceive a need for care (Table 5). After adjusting for relevant socio-demographic and health care service characteristics, risky alcohol and drug use were not associated with lifetime service utilization. However, risky weekly alcohol use was associated with lower current service utilization, while risky non-prescribed prescription drug use was associated with higher current service utilization.

**Table 4:** Weighted prevalence of perceived need for care and professional mental health service utilization for common mental disorders and risky substance use by sex and nationality (*n* = 981)

	Total	Male	Female	Swiss	Non-Swiss
	% (95% CI)	% (95% CI)	% (95% CI)	% (95% CI)	% (95% CI)
**Perceived need (lifetime)**					
ADHD alone	33.7% (24.9–42.5)	30.2% (17.8–42.7)	38.1% (25.9–50.2)	33.1% (23.2–42.9)	36.8% (17.7–55.9)
Anxiety alone	38.3% (29.1–47.5)	35.9% (22.1–49.8)	40.8% (28.9–52.7)	39.9% (29.6–50.1)	30.1% (10.6–49.5)
Depression alone	51.8% (45.6–58.1)	50.4% (38.9–62.0)	52.6% (45.3–60.0)	52.3% (45.1–59.5)	50.1% (37.4–62.9)
Anxiety and ADHD	60.7% (37.8–83.5)	60.1% (17.1–100.0)^a^	60.9% (34.5–87.3)	62.6% (38.0–87.1)	47.0% (0.0–100.0)^a^
Depression and ADHD	51.8% (38.2–65.3)	41.9% (16.4–67.4)	57.2% (41.9–72.6)	57.6% (41.8–73.3)	32.9% (9.9–55.9)
Anxiety and depression	61.2% (55.0–67.4)	56.2% (42.9–69.4)	63.2% (56.3–70.1)	61.7% (54.6–68.9)	59.5% (47.1–71.9)
ADHD, anxiety, and depression	65.2% (47.0–73.4)	65.7% (50.4–81.0)	65.0% (55.4–74.6)	65.4% (55.3–75.4)	65.0% (50.9–78.1)
Anxiety, depression, or ADHD	52.0% (48.8–55.3)	46.6% (40.8–52.4)	55.5% (51.6–59.3)	52.3% (48.5–56.0)	51.3% (44.8–57.8)
**Lifetime service utilization**					
ADHD alone	24.5% (16.5–32.5)	25.4% (13.7–37.2)	23.3% (13.0–33.6)	23.8% (14.8–32.8)	27.6% (10.2–44.9)
Anxiety alone	30.9% (22.4–39.3)	27.1% (14.3–39.8)	34.6% (23.6–45.7)	33.3% (23.8–42.9)	17.7% (1.7–33.7)
Depression alone	34.5% (28.6–40.3)	30.9% (20.2–41.6)	36.5% (29.6–43.4)	34.5% (27.7–41.2)	34.5% (22.5–46.4)
Anxiety and ADHD	33.8% (10.9–56.7)	43.6% (0.0–87.8)^a^	28.5% (3.3–53.7)	36.5% (11.3–61.7)	14.0% (0.0–42.7)^a^
Depression and ADHD	37.0% (24.1–49.9)	40.0% (14.81–65.1)	35.4% (20.8–49.9)	37.7% (22.4–53.0)	34.6% (11.3–57.8)
Anxiety and depression	45.3% (38.9–51.6)	30.8% (18.3–43.2)	50.9% (43.7–58.1)	46.3% (39.0–53.7)	41.9% (29.3–54.4)
ADHD, anxiety, and depression	53.6% (44.9–62.2)	42.0% (25.8–58.2)	59.5% (49.6–69.5)	54.2% (43.6–64.7)	52.3% (37.2–67.3)
Anxiety, depression, or ADHD	38.0% (34.8–41.1)	31.0% (25.6–36.4)	42.3% (38.6–46.1)	38.1% (34.5–41.7)	37.5% (31.2–43.9)
**Current service utilization**					
ADHD alone	9.4% (4.1–14.8)	8.9% (1.2–16.6)	10.1% (2.9–17.3)	9.4% (3.3–15.4)	9.7% (0.0–20.3)^a^
Anxiety alone	11.7% (5.7–17.6)	14.0% (4.2–23.9)	9.3% (2.8–15.9)	10.5% (4.2–16.9)	17.7% (1.7–33.7)
Depression alone	15.9% (11.3–20.4)	15.7% (7.1–24.2)	16.0% (10.8–21.2)	16.6% (11.3–21.9)	13.3% (4.8–21.8)
Anxiety and ADHD	6.1% (0.0–17.6)^a^	–^b^	9.3% (0.0–26.5)^a^	6.9% (0.0–19.9)^a^	–^b^
Depression and ADHD	15.4% (6.0–24.7)	12.3% (0.0–28.4)^a^	17.0% (5.6–28.5)	14.2% (3.5–24.8)	19.3% (0.0–38.9)^a^
Anxiety and depression	18.6% (13.7–23.4)	8.6% (1.6–15.6)	22.4% (16.4–28.5)	17.2% (11.9–22.6)	23.0% (12.0–33.9)
ADHD, anxiety, and depression	30.3% (22.5–38.2)	19.6% (7.0–32.2)	35.9% (26.2–45.6)	32.5% (22.8–42.2)	25.9% (12.6–39.2)
Anxiety, depression, or ADHD	16.8% (14.5–19.2)	12.7% (8.9–16.5)	19.4% (16.4–22.5)	16.3% (13.6–19.0)	18.6% (13.5–23.8)

^a^Confidence interval rounded to stay within possible values ^*b*^*no estimate*

**Table 5:** Factors associated with perceived need for care and service utilization among participants with common mental disorders (*n* = 981, weighted analysis)

	Perceived need (lifetime)		Lifetime service utilization		Current service utilization	
	OR (95% CI)	aOR (95% CI)^a^	OR (95% CI)	aOR (95% CI)^b^	OR (95% CI)	aOR (95% CI) ^b^
Risky alcohol use (at least monthly)	1.3 (1.0–1.7)	1.3 (1.0–1.7)	1.0 (0.8–1.3)	0.9 (0.6–1.2)	1.0 (0.7–1.4)	0.9 (0.6–1.3)
Risky alcohol use (at least weekly)	1.2 (0.9–1.8)	1.2 (0.8–1.8)	0.9 (0.6–1.3)	0.8 (0.5–1.2)	0.5 (0.3–0.9)	0.5 (0.3–0.8)
Risky cannabis use	1.8 (1.1–2.7)	1.9 (1.2–3.1)	1.2 (0.8–1.9)	1.0 (0.6–1.7)	1.1 (0.6–1.9)	1.0 (0.5–1.8)
Risky illicit drug use	3.4 (1.8–6.2)	3.3 (1.7–6.1)	1.4 (0.9–2.4)	1.1 (0.6–1.9)	1.0 (0.5–1.9)	0.8 (0.4–1.6)
Risky non-prescribed prescription drug use	2.2 (1.5–3.1)	2.1 (1.5–3.1)	1.8 (1.3–2.4)	1.4 (0.9–1.9)	2.6 (1.8–3.8)	2.2 (1.4–3.2)

^a^Adjusted for sex, nationality, language region, income, regional psychiatrist density

^b^Adjusted for sex, nationality, language region, income, regional psychiatrist density and perceived need for treatment

## Discussion

In this nationally representative study of young adults in Switzerland, around one-third of young women and one-fifth of young men met the criteria for at least one of the CMDs studied. Screening positive for at least one of the CMDs studied was more common in non-Swiss than in Swiss participants. Of all participants who met the criteria for CMDs, half did not perceive a need for treatment. Those screening positive for one or more CMDs reported more current suicidal ideation, lifetime suicide attempts, and lower mental health-related quality of life. Risky weekly alcohol among young people with CMDs was associated with lower current service utilization.

### Prevalence of CMD symptoms and risky substance use

Our current prevalence estimates for anxiety, depression, and ADHD in young Swiss adults are higher than those found in other international epidemiological studies [39, 40]. This could be due to geographical variation and cultural differences in the population examined and instrument used (i.e., a structured, in-person interview assessment as in CIDI vs. an online screening instrument). One could argue that the high prevalence values found in our study were due to self-report symptoms and the lack of a clinical diagnosis. However, all of the instruments for assessing CMDs have been used widely in clinical settings and demonstrated high specificity compared to the gold standard (82–99.5%) [23, 25]. This means that these instruments have a high probability of correctly identifying individuals without the disorder. In addition, we measured other mental health outcomes and found that those screening positive for one or more CMDs reported more current suicidal ideation and lifetime suicide attempts and worse mental health-related quality of life. It is also possible that, as has been observed in other countries [3, 15], the prevalence of these CMDs could be increasing in Switzerland, and many of the most widely referenced international prevalence studies have been performed over a decade ago.

Our prevalence estimate for depression can be compared directly with the PHQ-9 results from the Swiss Health Survey 2012 and 2017 [41] (the PHQ-9 was one of the only instruments on mental health covered in this survey). Our estimate of 17.7% is significantly higher than the estimate for 17–22 year-olds provided by the Swiss Federal Statistical Office at 10.9% in 2012 [41]. The values from the Swiss Health Survey 2017 were higher overall, including for the age group 15–24 at 12.3% for men and 13.9% for women [42]. Though estimates of the current prevalence of anxiety are not available for young adults in Switzerland specifically, international estimates are also lower than those found in our study [39, 40, 43].

At 8.7%, the estimate for the prevalence of ADHD in our study was in line with findings from the National Comorbidity Survey Replication–Adolescent Supplement in the U.S. and several recent cohort studies that reported estimates for adults at 8.1–12.2% [17–19]. In our study, ADHD symptoms were slightly more prevalent in women, though not significantly. This finding is supported by a recent growing body of literature that ADHD symptoms in adulthood may have an equal gender ratio [18] or that women may even be more likely to suffer from ADHD symptoms than men [19].

As in other studies [44], we observed sex differences in risky alcohol and drug use; however, this was not the case for monthly risky use of alcohol. Over the last years in Switzerland, men have reported less binge drinking, while women’s binge-drinking behavior has increased [45]. With more than a third of participants reporting monthly risky use of alcohol, it is possible that this behavior has become normalized among young Swiss adults.

We also observed differences in prevalence rates between Swiss and non-Swiss participants, with the rates generally higher in non-Swiss participants. These results overlapped those from a systematic review on the mental health of immigrants vs. the native population that found that immigrants suffered more often from CMDs [46]. While illicit drug use was similar between Swiss and non-Swiss participants, the non-Swiss participants reported lower levels of binge drinking, which could possibly be explained by different cultural or religious views on drinking.

### Treatment gap

Our study revealed a significant treatment gap among those who screened positive for a CMD. Given the high prevalence values found in this study, this represents a substantial absolute number of young adults in need of mental health care services that do not receive them with a correspondingly significant impact on public health. Although awareness of mental health issues is generally increasing in Western countries [47], the lack of perceived need could be due to low mental health literacy about clinical symptoms and the inability to seek help, which means that mental health disorders are still underdiagnosed and undertreated.

International studies have estimated the treatment gap at 50% or higher [8]; for example, the European Study of the Epidemiology of Mental Disorders (ESEMeD) found that 48% of people with mood, anxiety and substance use disorders severe enough to significantly interfere with everyday life received any formal healthcare [9]. In our study, we found that 38.0% (95% CI: 34.8–41.1%) of young adults with symptoms indicating current anxiety, depression, or ADHD had ever accessed care; this was close to the estimate of 35–37% found in the Swiss Health Survey 2012 [14]. The service utilization estimates from our survey are somewhat comparable to 12-month utilization rates of health care services for any mental disorder in several other high-income countries (between 23.9 and 62.1% for moderate to severe mental disorders) [48] at somewhere between 16.8% of participants with any of the three disorders currently using services and 38.0% who had ever used services.

### Frequent risky alcohol use associated with less service utilization

In line with previous studies [49, 50], we found that young adults with mental disorders who use alcohol are not more likely to perceive a need for professional mental health services, but are less likely to be currently using them. This could indicate a tendency to self-medicate in the absence of professional health care services since alcohol is a depressant that can temporarily relieve unpleasant symptoms of CMDs [49, 50]. However, while this practice can be effective at alleviating symptoms in the short-term, it is associated with risks in the long run [50]. In contrast, participants with mental disorders who use non-prescribed prescription drugs were more likely to currently use service. One possible explanation for this finding is that the participants with a mental disorder that used non-prescribed prescription drugs in our study were more likely to have depression and/or anxiety than ADHD, and the participants with these disorders tended to use services more than the participants with ADHD alone.

### Strengths and limitations

Our study was the first mental health survey of young adults across Switzerland. A major strength of our study was surveying a nationally representative sample obtained directly from the Federal Statistical Office. Our study was also subject to some limitations. First, our survey was conducted online instead of in person, and the instruments we used measured self-reported symptoms of mental disorders. Thus, although it was possible to grade participants’ endorsement of symptoms and classify them by disorder and severity using validated questionnaires with high sensitivity and specificity, participants lacked a clinical diagnosis. In addition, these self-reported symptoms may not reflect the impairment or level of functioning. However, we measured other mental health outcomes and found that screening positive for one or more CMDs was associated with worse mental health. Another limitation of our study was that measurement of mental health service availability was limited to psychiatrist density and did not account for the availability of other mental health professionals. Our study could also be subject to participation bias since those sampled who could not complete the survey in German, French or Italian are not represented in our study. Finally, we cannot rule out the possibility that the high prevalence found in our study was in part due to another source of participation bias, i.e., the fact that young adults with symptoms were more likely to participate in our study.

### Implications for practice and research

Because only around half of the people with a CMD reported lifetime perceived need for mental health care services, the first step in helping young adults with mental disorders to reach appropriate mental health care will involve interventions that increase knowledge and awareness about symptoms that could require professional mental health treatment. To uncover additional barriers to accessing care, we conducted some preliminary analyses (results not shown) and found that the most frequent major barrier was “wanting to solve the problem on [one’s] own.” It thus appears that the most common barriers to care in young Swiss adults reflect the same barriers found in other studies, namely lack of perceived need and the preference to manage the problem oneself [51]. To address the large treatment gap beyond this lack of perceived need, further research is needed to gain a detailed understanding of the barriers to care facing subgroups of young adults so that interventions can be tailored to address these specific barriers. Examples of such interventions include improving screening in primary health care, strengthening mental health service infrastructure, and disseminating psychological and mind-body therapies and self-care interventions [52].

Although we found that monthly binge drinking was widespread among young Swiss adults, those who binge drink more frequently could represent a specific group to be targeted by interventions to improve access to care since young adults with a CMD who reported risky weekly use of alcohol were less likely to be currently using services. These young adults may be unaware of the need to seek professional mental health treatment due to the fact that drinking alleviates their symptoms temporarily. If young adults could better recognize their own need and better access appropriate treatment, they might be less likely to use alcohol instead of utilizing mental health care services.

## Conclusions

The current prevalence of anxiety, depression, and ADHD symptoms in young Swiss adults is higher than many international estimates; as in other Western countries, the prevalence for these CMDs may be increasing. We should thus continue to observe this population and compare these results with other studies. Young Swiss adults’ low recognition of need and service utilization should be addressed through interventions to help individuals recognize moderate to severe symptoms in themselves and overcome barriers to reaching care. Young people with CMDs who frequently binge drink could particularly benefit from such interventions.

## Data Availability

The datasets analyzed in the current study are not publicly available due to the conditions specified in the data protection contract for this study.
